# Path Planning and Collision Risk Management Strategy for Multi-UAV Systems in 3D Environments

**DOI:** 10.3390/s21134414

**Published:** 2021-06-28

**Authors:** Blanca López, Javier Muñoz, Fernando Quevedo, Concepción A. Monje, Santiago Garrido, Luis E. Moreno

**Affiliations:** Robotics Lab, Universidad Carlos III de Madrid, Av. Madrid 30, 28911 Leganés, Spain; jamunozm@ing.uc3m.es (J.M.); fquevedo@ing.uc3m.es (F.Q.); cmonje@ing.uc3m.es (C.A.M.); sgarrido@ing.uc3m.es (S.G.); moreno@ing.uc3m.es (L.E.M.)

**Keywords:** multi-UAV systems, autonomous vehicle, fast marching, collision avoidance, path planning, velocity control, 3D Environment

## Abstract

Multi-UAV systems are attracting, especially in the last decade, the attention of researchers and companies of very different fields due to the great interest in developing systems capable of operating in a coordinated manner in complex scenarios and to cover and speed up applications that can be dangerous or tedious for people: search and rescue tasks, inspection of facilities, delivery of goods, surveillance, etc. Inspired by these needs, this work aims to design, implement and analyze a trajectory planning and collision avoidance strategy for multi-UAV systems in 3D environments. For this purpose, a study of the existing techniques for both problems is carried out and an innovative strategy based on Fast Marching Square—for the planning phase—and a simple priority-based speed control—as the method for conflict resolution—is proposed, together with prevention measures designed to try to limit and reduce the greatest number of conflicting situations that may occur between vehicles while they carry out their missions in a simulated 3D urban environment. The performance of the algorithm is evaluated successfully on the basis of certain conveniently chosen statistical measures that are collected throughout the simulation runs.

## 1. Introduction

Multi-UAV systems are attracting, especially in the last decade, the attention of researchers and companies of many different fields due to the great interest in developing systems able to operate in a coordinated manner in complex scenarios and to cover and speed up applications that can be dangerous or tedious for people. These can be found in the military, commercial or governmental domains and include search and rescue tasks [1,2], inspection of facilities [3], delivery of goods [4] and surveillance [5], among other fields.

Depending on the application, this coordination sought between the vehicles of the system can be approached from different points of view. In some cases, it may be necessary to coordinate the position and movements to maintain communication between UAVs in order to exchange some kind of information [6]. In other cases, this may be necessary to cover an area in a coordinated manner [7] and may also be needed to manipulate an object jointly [8]. In turn, this coordination can be studied at different levels: at a trajectory planning level, at a level of modeling and control of the vehicles, as a manipulation or communication between drones problem [9] or as a task allocation issue [10]. This work focuses on the path planning side.

One of the main concerns to be solved when developing an autonomous UAV system for the set of applications discussed above is its navigation, which is defined as the problem of taking a vehicle from an initial point to a final point, safely avoiding collisions with obstacles present in the environment and with other UAVs, all of this in an autonomous way. In general, this problem is decomposed into two phases [11,12]: generation of trajectories from the starting point to the goal for each vehicle (i.e., global path planning) and local collision avoidance. In the first phase, there is already an obstacle-free path planning (of those obstacles that are known from the environment), while the second phase focuses on a refinement of those trajectories to deal with collisions that cannot be avoided or predicted beforehand.

The trajectory to be planned for the drones to follow must satisfy these conditions:

- Respect the geometry of the environment.
- Ensure a safe distance of the vehicle with respect to objects.
- Minimize the risk of collisions.
- Provide a route that is navigable by the vehicle, i.e., that respects its kinematic restrictions.

Different planning algorithms have been applied to solve this trajectory generation problem. For example, the RRT algorithm is used in [13,14], whose resulting trajectory is subsequently smoothed so that it is achievable by vehicles. On the other hand, A* is used in [15], where it is also used as a basis to avoid conflicting situations with other UAVs in the collision avoidance stage. An example of the use of PRM as a possible solution to this problem is found in [16], where the authors discretized the space in voxels, and, once the graph connecting the nodes of the free space is obtained, A* is used to trace the optimal route. Shanmugavel et al. [17] dealt with this global path planning phase by means of combinations of straight Dubin trajectories connected by clotoid curves. Borrelli et al. [18] treated the problem as an optimization problem and compared its solution using nonlinear programming algorithms (NLP algorithms) and integer linear programming algorithms (MILP algorithms). Finally, Galvez et al. [19] detailed the method to be followed to generate a trajectory from an initial point to an end point, avoiding the known obstacles of the environment, for a quadrotor by implementing a genetic algorithm.

In this work, the algorithm used to plan the path of each vehicle from its starting depot to its destination position is Fast Marching, specifically, its square form (Fast Marching Square). This method, in addition to resulting in the shortest route in distance as well as the safest, enables the addition of the time variable to the solution, giving information about the speed of the vehicle at each waypoint of the trajectory. This allows a greater knowledge, control and thus variety of design options for the solution. Furthermore, our RoboticsLab group has already worked on the implementation of this algorithm in 3D environments [20,21], although for a single vehicle. This work faces the first step towards extending its application to multi-UAV systems.

In the second planning stage, in order to prevent a collision from occurring, the vehicle control system has to be able to cope with the following tasks [22]:

- Detect possible collision threats.
- Analyze the actual collision probability and define decision criteria.
- Implement the collision avoidance algorithm.

To do so, it is mandatory that the vehicle has some information about the trajectory or position of the obstacle to avoid. This process is similar to the one that manned aerial vehicles have to perform when navigating in automatic mode, and, therefore, it may seem natural to use the same techniques (already studied and developed) in UAVs. In the case of TCAS, it is designed mainly for paired-manned-aircraft encounters [23,24]. The authors of [25,26] emphasized that using the same techniques in UAVs as in manned vehicles can be unfeasible because the set of UAVs is much more heterogeneous (in relation to vehicle characteristics, control mode, UAV type, etc.). Freitas [27] also pointed out that there are environments in which the telemetry required by these systems is not available, such as in indoor environments. For its part, some solutions for collision avoidance of UAV systems implementing ADS-B technologies are found in the literature [22,28] to estimate the current position of vehicles and share these estimates with others. It should be noted that ADS-B would only provide information from other friendly vehicles and not from any other type of obstacles. Therefore, researchers have focused on creating collision avoidance algorithms for UAV systems based on sensor and vision systems mounted on the drone as a mean of interaction with the environment. These systems can be cameras, infrared sensors, laser scanners (LiDAR) or radar systems, among others. It may also happen that UAVs work cooperatively and, when in communication range, share information about obstacles that they have learned about individually.

Thus, multiple and varied solutions have been proposed to address the collision avoidance problem. Checking the literature, there exist multiple classifications that aim to encompass all these varied solutions. Thus, geometric algorithms can be found, where the main strategy is known as velocity obstacles [11,29], which is defined geometrically as the set of velocities to avoid as they would lead to a collision with some other element, at some point in time, provided that the current velocity of this other element does not vary [30]. Other options consist on algorithms based on vision methods, including those focused on working with sensor parameters such as the field of view [31] or based on the UAV parameters such as the relative azimuthal angle of the vehicle [32,33]. Pure image processing techniques [34,35] are also a commonly used alternative. Likewise, strategies based on path planning algorithms [36,37] and potential fields [38,39], previously discussed, can be found, as well as algorithms based on optimization problems [40,41,42]. Finally, outside any other category, there are those custom strategies that, without following any specific methodology, aim at developing a collision avoidance strategy as simple as possible to keep the computational load at the lowest, an issue that can be hindered in more complex strategies (in terms of management and resolution calculation of conflicts) [43,44,45,46,47]. These strategies rely on either altitude or lateral deviation of vehicles through simple decision processes. It should be noted that many of the strategies found in the literature related to the collision avoidance problem are not aimed at multi-UAV systems (just restricted to one or few vehicles), directed to open field spaces with few obstacles and still in the experimental phase or even limited to a theoretical approach for the time being.

As far as this work is concerned, it is the latter strategies proposing simpler resolutions that have inspired the one implemented in this project. The objective is to develop the simplest possible collision avoidance strategy based on the speed control of the vehicles of the multi-UAV system and to study through simulations its capacity to prevent and minimize the number of conflicts that could occur while the drones complete their missions and to resolve those that could not be avoided. A low computational cost facilitates the scalability of the method to a larger number of vehicles and its future implementation in real applications, becoming a strong contribution of our proposed strategy.

The application that has inspired this work belongs to the commercial field, in particular, the task of parcel delivery using UAVs. This initiative constitutes the Prime Air project of the Amazon company [48], which aims to offer its customers a super-fast delivery of 30 min or less from the time they place their order. In this way, the developed planning and collision avoidance algorithm is applied to a simulated environment that resembles an urban setting. This work will mean a first approach of our group to the real use of autonomous multi-UAV swarms in cluttered urban environments, currently not available.

Furthermore, the usefulness of the algorithm developed is broad and general, so it can be applied to other tasks. In summary, the objective is to get a set of drones, from an exit point of the environment (referred to as depot), to their respective final locations. After this process, one could either implement the delivery task mentioned above or start a routine to cover any of the other applications previously highlighted. As for this work, the execution is limited to the arrival at the end point. Once the mission is completed, the drone returns to its starting point, to begin a new mission.

The rest of this paper is organized as follows. Section 2 details all the characteristics of the proposed path planning and collision risk management strategy and its implementation. Section 3 presents the statistical measures collected to evaluate the performance of the strategy. The resulting data are conveniently analyzed. Finally, Section 4 provides the main conclusions drawn from the development of this work.

## 2. Description of the Approach

As detailed above, this work aims to develop a trajectory planning and collision avoidance strategy for multi-UAV systems in 3D environments. This is purely done on the basis of experiments carried out in simulated environments, using MATLAB software. In this project, drones are considered as a point-size mobile occupying a certain position in the free space of the environment. The description of the developed algorithm is divided into three large sections for the sake of clarity, which correspond to the three main stages that make up the strategy: the representation and description of the environment phase, the trajectory planning using Fast Marching phase and the implementation of the designed collision avoidance phase. It is important to note that the terms UAV and drone are used in this work equally.

### 2.1. Representation and Description of the Environment

Among the applications aimed at multi-UAV systems, as highlighted above, the one that has inspired this work corresponds to the commercial field, in particular, the task of parcel delivery in urban environments through the use of these swarms of vehicles. Therefore, the missions described for this work for each vehicle to fulfill consist of going from its assigned departure point to a certain target point in the environment. After completing this journey, the drone returns to its starting point to begin a new mission.

These starting points are named and referred to throughout the text as depots. Two depots are defined in this work, dividing by half the number of drones that leave each depot from the total number of drones to be planned.

**Figure 1:**
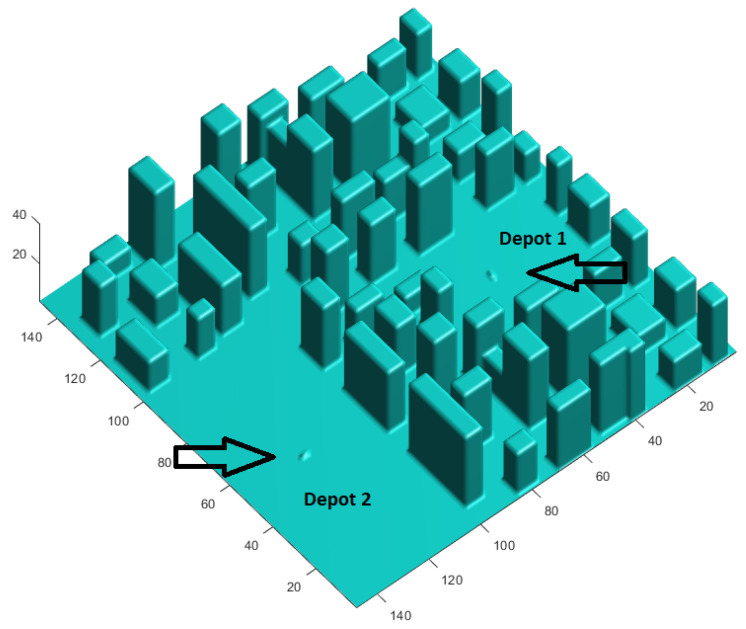
Simulated urban environment used for the tests performed in this work.

The environment over which the flight paths for the drones are planned consists of a 3D scenario that resembles an urban environment with buildings of different heights. The environment is modeled as a 3D occupancy matrix and can be visualized in Figure 1. The resolution of the map is considered to be 3 m, in the three dimensions of space, and its dimensions are 150 × 150 × 50 cells.

The figure shows the two depots reserved for the takeoff and landing of the drones. Around each of them, a safety distance is guaranteed for vehicles to take off. This helpa to manage in a special way the transit of drones in this area, which can be conflictive as it is prone to a high concentration of drones flying at very short distances. This procedure is explained below.

### 2.2. Trajectory Planning Using Fast Marching Square

Once the simulated environment to work on has been presented, the UAV trajectory planning phase begins. As previously mentioned, the UAVs will fly from an initial point (its corresponding depot) to an end point. In this work, these end points are randomly chosen positions in the environment free of obstacles (a condition to be expressly verified). Of course, these positions could also be fixed at will. Given the trajectory, the drone will follow it until it reaches the end position, at which point the trajectory back to the depot is planned.

Both trajectories, the one to the target point and the one back to the starting point, are obtained using the Fast Marching Square algorithm. The Fast Marching Method (FMM) is a method used to numerically solve the Eikonal equation (Equation (1)), which models the propagation behavior of a wave (e.g., a ray of light) by the arrival time of that wave at any point in space. Originally, it was proposed by Sethian in 1996 [49]. In the equation, ρ represents any point in the environment, T(ρ) is the time it would take for the wave to arrive from the initial point to the ρ point and F(ρ) is the velocity of the wave propagation. As can be deduced, at the origin point of the wave expansion, one has that T(0)=0.

(1) $$1=F\left(\rho \right)|\nabla T\left(\rho \right)|,\rho \subset R^{N}$$

According to Fermat’s principle, a wave propagating between two points in space through a material medium always propagates along the path that takes the least time. Therefore, Fast Marching is of great interest as a planning algorithm given the similarity between the two problems.

Thus, if we want to calculate the optimal path in terms of time between two points in a given environment, considering that the starting point is a light wave source and applying FMM on the obstacle-free space of the environment, we can obtain the time it would take to reach any point from the point of origin. Then, from any point in the environment and by means of gradient descent techniques, it is possible to compute the trajectory that leads to the origin point by the optimal path.

In addition to our own work previously mentioned, other researchers have also applied this method to the path planning problem [50,51,52,53,54,55].

**Figure 2:**
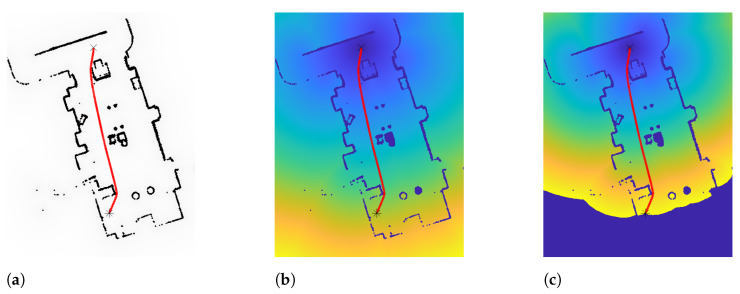
Stages of the FMM. (**a**) Binary map representing F(ρ) obtained through SLAM navigation. (**b**) Representation of the arrival times T(ρ) of the wavefront. (**c**) Representation of wavefront propagation stopping when reaching goal point. Final trajectory is represented on every map.

An example of the implementation of FMM in a two-dimensional environment can be seen in Figure 2. As can be observed, the wave propagation velocity is simply discretized to zero values inside the obstacles and one (total velocity) in free space.

In contrast, the Fast Marching method in its original form has some drawbacks. For example, as the trajectory obtained is the optimal in time and distance, it tends to get too close to obstacles, which would be dangerous for a vehicle to follow. Similarly, the trajectory may not be sufficiently smooth and therefore achievable by the vehicle given its kinematic constraints. These two problems are solved in the variant known as Fast Marching Square (FM2), proposed by our group [56,57]. FM2 achieves this by playing with the values of the propagation velocity in free space F(ρ) and making them depend, for each point, on its distance to the nearest obstacle: points farther away from obstacles will allow the propagating wave to have a higher velocity than points closer to obstacles. The wave will tend to follow paths with higher velocity values, and thus safer ones.

**Figure 3:**
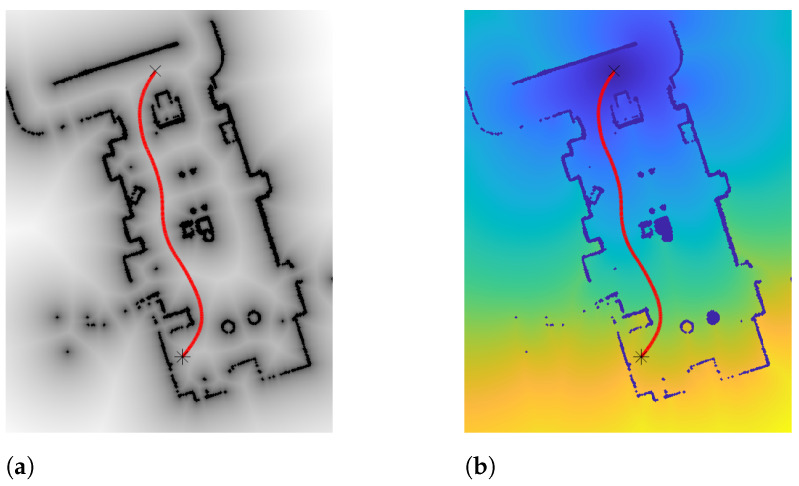
Example of FM2 implementation. (**a**) Velocity map saturated at a value of 0.6. (**b**) Representation of the arrival times T(ρ) in the second FMM stage. The final trajectory is represented on both maps.

This modification of the original method consists of applying the FMM twice. Firstly, it is applied from each point in the environment that constitutes an obstacle. As a result, each cell in space acquires a time value T(ρ) which symbolizes the time required to reach that cell from the nearest obstacle. These values are rescaled to the interval [0,1]; therefore, the resulting map can be represented in gray scale. This map can be seen as a map of velocities or viscosity: higher values of T(ρ) indicate greater distances to obstacles; thus, at such points, the vehicle will be able to acquire higher velocities. In the second stage, these values of T(ρ) obtained in the previous stage act as propagation velocity values F(ρ). Similar to the original FMM, the trajectory is finally obtained by gradient descent techniques. The velocity map obtained in the first stage can be saturated to consider higher velocities at shorter distances. An example of the implementation of FM2 in a two-dimensional environment can be seen in Figure 3.

The complete description of this algorithm is explained in detail in [58]. As a summary, the main advantages of applying FMM (or its FM2 variant) to planning problems are summarized below:

- It is a complete planning algorithm, i.e., it always finds the solution to the problem, if it exists.
- It completely eliminates the problem of local minima. A local minimum would imply that such point would be assigned a lower T(ρ) than neighboring points closer to the wave source, which is impossible considering that F(ρ)≥0∀ρ.
- The trajectory obtained between two points in space is the optimal in both time and distance. In the case of FM2, a smooth and safe trajectory is obtained, which remains sufficiently optimal.
- It is an algorithm of linear complexity order O(n), where n represents the total number of points of the considered mesh, which makes it computationally faster than other planning algorithms whose complexity order is, for instance, exponential (e.g., the A* algorithm) or asymptotic (e.g., the RRT algorithm).

Having explained the general operation of the Fast Marching and Fast Marching Square algorithms, hereafter some details about the direct application of the Fast Marching Square method in the designed algorithm are highlighted. To begin with, it should be noted that the planning algorithm has been developed based on Gabriel Peyre’s Fast Marching MATLAB toolbox [59].

The implementation of this algorithm requires three parameters: starting point coordinates, final point coordinates and a map of the environment to plan on. As previously mentioned, in the outbound missions, all drones depart from their respective depot (half of the UAVs depart from one of the two defined depots and the rest from the other). On the other hand, the destination point is randomly generated, having guaranteed that it corresponds to a point in the free space and is outside the depot exclusion zones (detailed below). For the return mission to the depot, the destination point is the depot itself and the starting point is the end point of the outbound mission trajectory, which coincides with the current position of the drone.

After defining the starting and end points of the trajectory, it is important to highlight the characteristics of the map on which the trajectory is planned. As mentioned above, the baseline is the binary occupation map of the environment. FM2 is applied to this map which, as detailed above, consists of applying the FMM algorithm twice. However, the first application of FMM can be replaced in practice by any function that calculates the Euclidean distance transformation of the original binary map.

Once this first step is completed, the aforementioned viscosity matrix or map is obtained, which, in this case and compared to the previous example figures, consists of three dimensions. One of the strengths of the Fast Marching method is the relative ease with which constraints can be incorporated in the planning process of the trajectories. It is sufficient to define a new matrix, of the same dimensions as the working environment, where the cells corresponding to the areas to be restricted provide low velocity values, and then add (just by matrix multiplication) these restrictions to those already imposed in the previously obtained viscosity maps.

Thanks to this feature, two new restrictions are added to the already considered restrictions by distance to obstacles. Both are aimed at limiting cases of conflict, understanding these as risk situations in which two or more vehicles are at a distance less than a certain established safety distance and, therefore, can lead to a collision. The first measure prohibits drones from a given depot from flying over an opposing depot. The second establishes flight levels at which UAVs tend to fly.

Specifically, this work sets four different flight levels with respect to ground level: flight level at altitude 9, flight level at altitude 15, flight level at altitude 21 and flight level at altitude 27. As can be appreciated, the flight levels are defined with a height difference of 6 cells. This is considered enough since, as specified in the section related to the collision avoidance stage, it is established that for a conflict to exist two UAVs must be at a distance of less than 12 in the horizontal plane and 5 in altitude.

To establish these flight levels, Equation (2) is specified below, which calculates the viscosity (or velocity value) assigned to each of the environment cells in relation to their height, for each map height:

(2) $$\mathrm{Viscosity}\;\mathrm{value}\;\mathrm{according}\;\mathrm{to}\;\mathrm{height}=\frac{1}{0.5\times {\left(|zref-k|\times 0.005+0.01\right)}^{Es}}$$

where *zref* is the flight level to be set, *k* is the map height whose viscosity value is being calculated and *Es* is the aggressiveness factor according to which the flight level is set. Lower values of Es imply lower aggressiveness, i.e., the UAV is less forced to fly at the set flight level, especially if Es is less than 1. This equation is applied to an empty matrix of the same dimensions as the occupancy matrix of the environment. The resulting matrices are considered along with the previously computed velocity matrix related to distances to obstacles. It is important to note that, in order to be used in the second FMM application of FM2, these matrices must be rescaled to the interval [0,1].

The way to assign a specific flight level to a given mission is detailed in the following section. It should be highlighted that this assigned flight level is followed by the UAV as far as possible. In the trajectory stages where it needs to modify its altitude to reach the desired points in space, the vehicle can fly at a different altitude than the one assigned.

As for the characteristics of the trajectories designed, it was decided that, again to avoid conflicts in the area near the depots due to crossings where the flight level is not maintained for being in the takeoff/landing zone, the UAVs in this area should only fly perpendicular or parallel to the horizontal plane, so that the flight level is guaranteed. Once the complete path is obtained, having applied for the second time FMM to the viscosity map and by gradient descent techniques using the corresponding start and end points, it is smoothed. To do this, each calculated coordinate of the path is replaced by the average of its own value and the value of the trajectory coordinates placed before and after.

**Figure 4:**
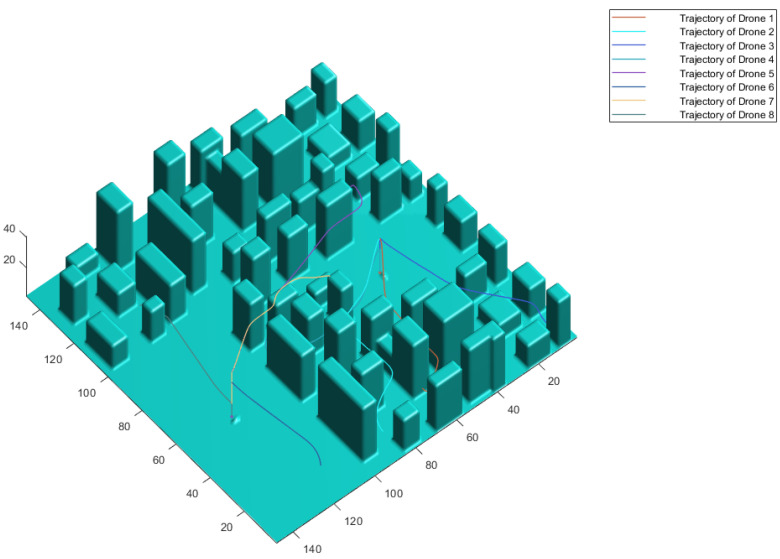
Representation of the trajectories calculated with FM2 on the simulated environment.

**Figure 5:**
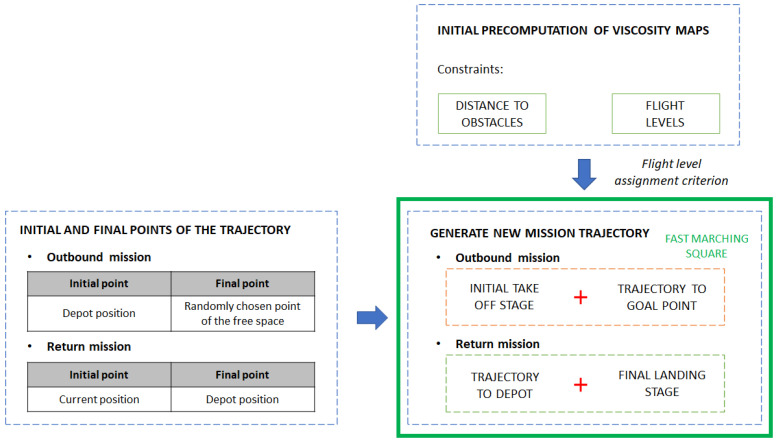
Schematic representation of the path planning stage.

At the end of this Fast Marching Square trajectory planning phase, the first outbound trajectories are obtained and represented in the simulated environment map, as shown in Figure 4 for a simulation of a swarm with eight drones. The two sections that compose each path can be appreciated, and it can also be distinguished that, in the intermediate part of each path, it keeps parallel to the ground plane, respecting the assigned flight level. This whole path planning stage is summarized graphically in Figure 5.

### 2.3. Collision Risk Management by Vehicle Velocity Control

After this path planning stage, the collision avoidance phase begins. This is carried out iteratively in a loop. The conflicts considered in this work are those that may occur between vehicles. Therefore, this implies that they work cooperatively (they are not intruding vehicles) and their position can be known. Therefore, conflicts with dynamic elements of the environment are discarded and static elements have already been considered in the first phase of trajectory planning.

Within this phase, two categories are distinguished: preventive strategies and resolution strategies. Preventive strategies include those that, as their name suggests, prevent and try to reduce the number of conflicts that occur between UAVs. The conflicts that are not eliminated by these measures are the ones to be solved by the resolution strategy. Before starting any explanation, it is worth mentioning that in this work drones are identified by an assigned index (as shown in Figure 4), but this does not establish any sort of priority over other vehicles.

Preventive measures include those that have been discussed so far: flight levels imposition and special management of the area near the depots. Furthermore, a safety distance check (similar to that carried out in the depots) to start the return flight back to the depot is also performed. Regarding flight levels, their implementation has been sufficiently explained in the previous section. For this part, it is necessary to clarify how each UAV is assigned to a specific flight level. The flight level depends on the direction of flight, more specifically on the angle formed by the vector joining the initial and final point of the trajectory (in the direction of the trajectory, and according to the *xy* coordinates) with the *y*-coordinate axis of the map. In this way, four angle ranges are distinguished, each associated to a flight level. This assignment is summarized in Table 1. As can be appreciated, this method is aimed at eliminating the conflicts of drones flying in opposite directions, which are, according to the resolution strategy implemented and explained below, the most problematic. Thus, drones flying at opposite angles are separated, a priori, by 12 levels in altitude.

The second preventive measure is related to the UAV traffic management in the areas near each depot, which serve to specially control the drone flow in this area prone to a high concentration of drones flying at very short distances. The rule governing traffic in these so-called exclusion zones is that no vehicle can take off and begin its new outbound mission if any other vehicle is flying closer than a safety distance, which is fixed to a value of 30 cells in the horizontal plane. This means that drones that are in the depot waiting to take off have to wait in the depot to do so when allowed.

Thus, this traffic management creates a “waiting queue of drones” that want to fly over the exclusion zone (one for each depot). At the beginning of each iteration of the control loop, it is analyzed, for each depot, if the exclusion zone is free of vehicles (according to this rule that has been established) so that, if there is any drone in the queue, it can start its flight. If so, the first vehicle in the queue starts its mission and is removed from the waiting list.

A similar safety distance check is carried out when a vehicle ends its outbound mission and wants to begin its return mission to the depot. In this case, it is considered that, once the vehicle reaches its goal point, it finds itself in a safe place. No collision risk is contemplated for these vehicles. Then, to start the pertinent return mission, it must be guaranteed that no other vehicles are flying in the vicinity (the safety distance considered is the same as for the depots exclusion areas). No “waiting queue” is generated on these occasions.

Therefore, it could be said that vehicles can be classified into two categories: Those currently flying and those waiting to do so, either in their depot or in their goal point. These rules will help to control and reduce the number of potential conflicts between flying vehicles.

Once this first stage of the iterative control loop of the collision avoidance phase has been completed, the conflict resolution strategy begins. The first thing to do is to identify those conflicts. This is done in a simple way through a distance-between-vehicles check. In order of the identifying index assigned and by pairs of drones, the distance to the other drones of higher indexes is analyzed. An important step of the conflict resolution strategy is implemented herein. First, it is studied if the two vehicles being analyzed are closer than a fixed distance value. As for this work, it is 25 cells. If so, their remaining trajectories are further examined. If not, no potential conflict is detected for now between those vehicles. This preliminary check allows a wider margin of maneouver, as conflicts can be identified early.

In case a further analysis of the trajectories is required, the distance between both of them is evaluated for every waypoint. This distance is studied independently both in the horizontal plane and in height. Once the analysis is completed, a conflict is considered to exist if both trajectories are nearer than a given safety distance, at any point. For the horizontal *xy* plane, the safety distance is set as 12 cells, while on the *z*-axis the safety distance is 5 cells. The imposition of flight levels allows setting a less restrictive distance on the vertical axis. If the trajectories do not meet this requisite, being farther from each other, no potential conflict is considered for now between those vehicles.

**Figure 6:**
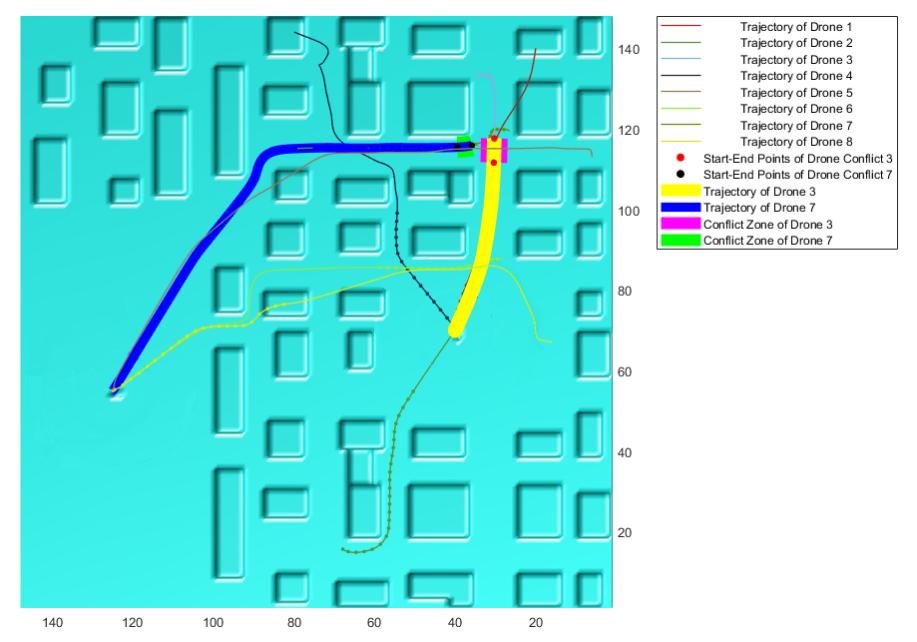
Example of the representation of information of a new conflict detected.

According to these conditions, if a new case of conflict is detected, its resolution commences. First, the sections of both trajectories in conflict with each other are identified. This information is visually represented on the simulated working environment, as shown in the example of Figure 6. In magenta and green, the conflicting sections are emphasized, delimited by red and black dots. The remaining trajectories of both drones are highlighted in yellow and blue, respectively. The already traveled path and the current position of the drones are represented through points of the same color of the corresponding trajectory, in a thicker line.

After this first analysis, the mode of resolution of the conflict is identified. First, the broad outline of the conflict resolution strategy implemented must be known beforehand. This is kept as simple as possible, in order to achieve a low computation time and a useful strategy at the same time. Its principle of operation is as follows: one of the drones in conflict stops its flight, while the other one continues its trajectory normally. This is the case if both vehicles were, just before the conflict, in motion. If this is not the situation, both vehicles stop their flight.

As previously mentioned, flight level imposition helps to avoid conflicts between vehicles flying closer and in opposite directions. Furthermore, the early analysis of potential conflicts detailed above allows to further guarantee that this does not happen, for instance, in the trajectory stages where the flight levels cannot be maintained. For these reasons, this simple strategy is found to be enough to avoid collisions between vehicles, making trajectory deviations not mandatory and thus eliminating the computational load required for path recalculations.

The criterion to decide which vehicle continues and which vehicle stops its flight is determined by the type of conflict and depends on:

- Which vehicle is found flying behind the other, in the case both are flying in the same direction.
- Which vehicle is found flying nearer to the closest point between their trajectories, in the case both are flying in opposite directions.

In both scenarios, it is the vehicle highlighted above which is considered as the non-priority one and is commanded to stop its flight. It is interesting to note that this identification of type of conflict and assignment of preferences is only performed in the iteration in which the conflict is detected.

Once the type of conflict has been analyzed, we proceed to its actual resolution. It is important to emphasize that the movement or not of the drones is given by their speed. This value is considered as a percentage of 1. Thus, a vehicle with speed 1 would fly at 100% of its speed, while a vehicle with speed 0 would be stopped. In other words, vehicles at total speed move each iteration a step farther on its planned path, whereas vehicles with 0 velocity remain at its current position, hovering for instance. In general, drones have one of these two speeds: zero speed for those non-priority vehicles in some active conflict as well as for those waiting to take off and full speed for the rest of the UAVs. However, when a new conflict is identified, a drone that has to reduce its speed does so by 0.5 each iteration. Similarly, when the active conflicts are resolved and a drone can resume its speed, it increases by 0.5 every iteration. In the future, if the algorithm is to be applied to real drone systems, these values will have to be adjusted to the kinematic characteristics of the drones.

An active conflict between two vehicles is considered to be finished if the very same conditions established to detect new conflicts are no longer met.

**Figure 7:**
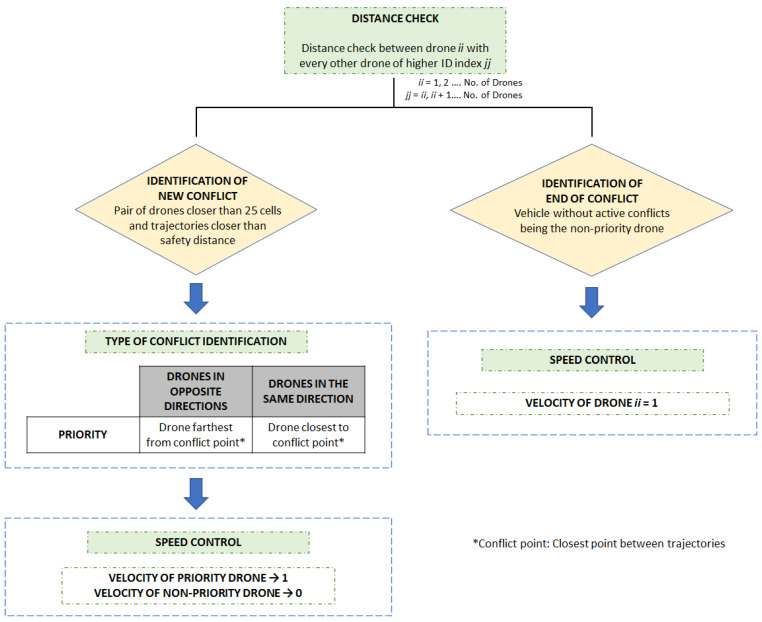
Schematic representation of the collision risk management stage.

It must be contemplated that a drone can be in conflict at the same time with more than one vehicle. Therefore, after analyzing the situation for all possible pairs of drones, each one always takes the speed value required in order to respect any possible conflict that has been detected. This is conveniently expressed by an arrow in Figure 7, which summarizes the collision risk management stage. Different information regarding the development process of conflicts is collected through statistical measures, which are detailed hereinafter.

The whole working process of the developed algorithm is expressed through pseudocode in Algorithms 1–4, to facilitate its understanding.
**Algorithm 1** Routine of the complete developed strategy1:Wo← Occupation matrix of the simulated environment2:W_array← Viscosity maps computed from Wo. Constraints: Distance to obstacles and flight levels.3:Define number of drones4:Define the position of the two depots5:Depot assignment: Half of drones to one depot and the other half to the other6:iteration_index← 07:**while**iiteration_index < 7000 **do**8:    depot_queues← Queues of drones waiting to take off in depots9:    **for** every drone **do**10:        **if** drone ends its current mission **or** iteration_index == 0 **then**11:           drone.path←Generate_mission(drone.position,drone.depot_position,W_array)12:           drone.velocity← 013:        **end if**14:        **if** drone is waiting to start new mission **then**15:           drone.velocity←Take_off_distance_check(drone.ID,depot_queues)16:        **end if**17:        **for** every drone_of_higher_ID **do**18:           (v1,v2) ←Check_conflicts(drone, drone_of_higher_ID)19:           drone.velocity←v120:           drone_of_higher_ID.velocity←v221:        **end for**22:        **if** drone without conflicts being the non_priority vehicle **and** not landed **then**23:           drone.velocity← 124:        **end if**25:    **end for**26:    iteration_index←iteration_index + 127:**end while**

**Algorithm 2** Trajectory planning using Fast Marching Square
  **function**Generate_new_mission(dronePos, depotPos, Warray)2:  **if** New outbound mission **then**        Initial point←depotPos4:        Final point← Randomly chosen point of free space    **else**6:        Initial point←dronePos        Final point←depotPos8:    **end if**    Assign flight level according to flight direction10:  *W*← Viscosity map from Warray according to flight level  Determine intermediate point at depot and flight level height12:  **if** New outbound mission **then**        Calculate takeoff stage of the trajectory using FM2 and *W*14:        Calculate the rest of the trajectory to goal point using FM2 and *W*  **else**16:        Calculate landing stage of the trajectory using FM2 and *W*        Calculate the rest of the trajectory to depot using FM2 and *W*18:  **end if**  path← Composition of the complete trajectory20:  **return** path
**end function**



**Algorithm 3** Distance check function to ensure a safe takeoff
     **function**Take_off_distance_check(droneID, depot_queues)      **if** (New outbound mission **and** droneID first in its corresponding depot_queues) **or** New return mission **then**3:              **if** no other drones flying near **then**                    new_velocity← 1              **else**6:                     new_velocity← 0          **end if**9:          **return** new_velocity
**    end function**



**Algorithm 4** Conflict management stage function
 **function**Check_conflicts(drone, drone_of_higher_ID)     safety_distance← 12 cells in horizontal plane and 5 cells in vertical axis      **if** Drones closer than 25 cells and trajectories closer than safety_distance **then**4:          **if** New conflict **then**                Assign conflict priorities to both drones          **end if**          priority_drone.velocity ← min(priority_drone.velocity,1)8:          non_priority_drone.velocity ← 0      **end if**      **return** drone.velocity, drone_of_higher_ID.velocity
**end function**



## 3. Results

Once the path planning and collision avoidance algorithm for a multi-UAV and multi-depot system, specifically designed for its application in urban environments, was designed and displayed, a series of statistical measures was defined to test its performance. The statistical measures collected were chosen in order to allow a complete evaluation of all the functionalities implemented in the code. These are listed and detailed below:

- For each mission completed by each vehicle, the **length of the trajectories** corresponding to the outbound and return missions is registered.
- The **number of totally completed missions** performed by the set of drones is recorded.
- In addition to the previous collected measure, the **number of missions completed by each vehicle** is recorded.
- **List of conflicts.** For each conflict that has occurred throughout the whole execution, a series of measures is analyzed: pair of drones that have participated in the conflict, duration of the conflict and smallest distance given between both vehicles (both in the *xy* plane and in the *z*-axis).
- **Number of conflicts occurred for the set of drones.**
- **Characteristic mission time measures.** For each mission completed by each vehicle individually, eight measurements are recorded: time spent waiting in depot to start outbound mission, time spent stopped in flight due to conflicts in outbound mission, total duration of outbound mission, number of conflicts encountered in outbound mission, time spent waiting at the goal point to begin return mission, time spent stopped in flight due to conflicts in return mission, duration of return mission and number of conflicts in return mission.
- **Other time measures.** The time required for each iteration run is also recorded. The time spent in the routine responsible for moving the drones and updating their trajectories is also taken into account.
  The aim of these measurements is to calculate the real computation time of the algorithm iterative loop, discarding as much as possible the time spent in calculation processes that belong to the simulation itself and are not an essential part of the path planning and collision avoidance stages.

Of all the measures collected, the only one that is not obtained directly from the simulation is the real flight time/useful time ratio, which is used to study the relationship between the flight time spent in each mission against the time the drone has actually been in motion and not stopped due to conflicts. The way to obtain this ratio from the previous measurements is therefore described in Equation (3), where the word “Time” is abbreviated for convenience by the letter T. Therefore, the ideal outcome for this measure would be a value of 1, meaning the vehicle was in motion during its entire flight time.

(3) $$\frac{\mathrm{Real}\;\mathrm{flight}\;T.}{\mathrm{Useful}\;\mathrm{flight}\;T.}=\frac{\mathrm{Total}\;\mathrm{flight}\;T.-T.\;\mathrm{waiting}\;\mathrm{to}\;\mathrm{take}\;\mathrm{off}}{\mathrm{Total}\;\mathrm{flight}\;T.-T.\;\mathrm{waiting}\;\mathrm{to}\;\mathrm{take}\;\mathrm{off}-T.\;\mathrm{stopped}\;\mathrm{in}\;\mathrm{flight}}$$

These measurements were collected over the course of three runs of the algorithm, of 7000 iterations each. Furthermore, the tests were carried out for swarms with different numbers of drones,: 4, 8, 12, 16, 20, 24 and 28. As already mentioned, two depots were used for all simulations. For reference, the tests were performed on a computer with 16 GB RAM and an AMD Ryzen 5 5600X CPU @ 4.12 GHz. Simulation graphic representations previously shown are turned off during data gathering.

**Figure 8:**
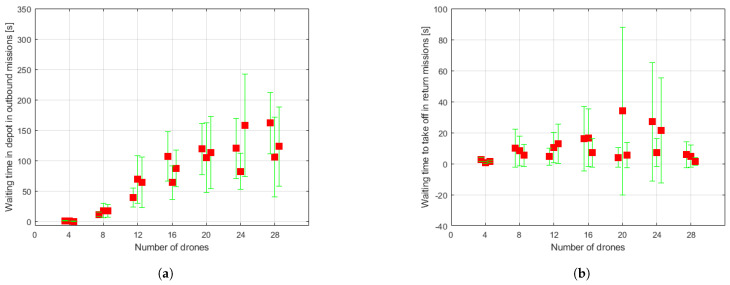
Statistical measures collected through simulations. Red squares represent mean values, while green ranges indicate mean absolute deviation. (**a**) Waiting time in depot in outbound missions. (**b**) Waiting time to take off in goal point to begin return mission.

**Figure 9:**
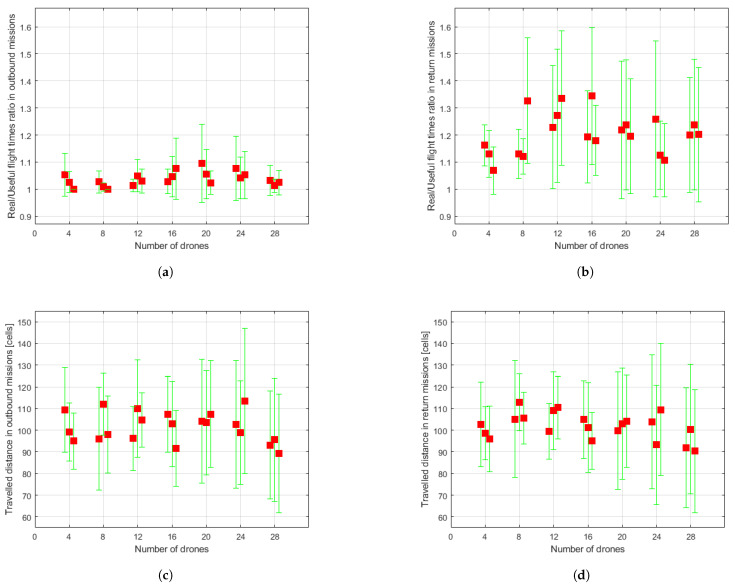

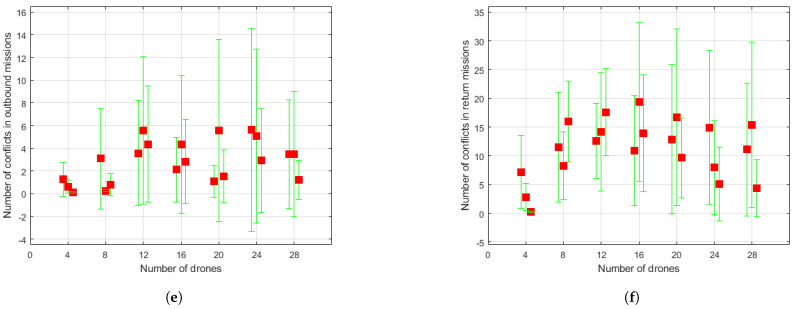
Statistical measures collected through simulations. Red squares represent mean values, while green ranges indicate mean absolute deviation. (**a**) Real flight time/useful time ratio in outbound missions. (**b**) Real flight time/useful time ratio in return missions. (**c**) Traveled distance in outbound missions. (**d**) Traveled distance in return missions. (**e**) Number of conflicts in outbound missions. (**f**) Number of conflicts in return missions.

**Figure 10:**
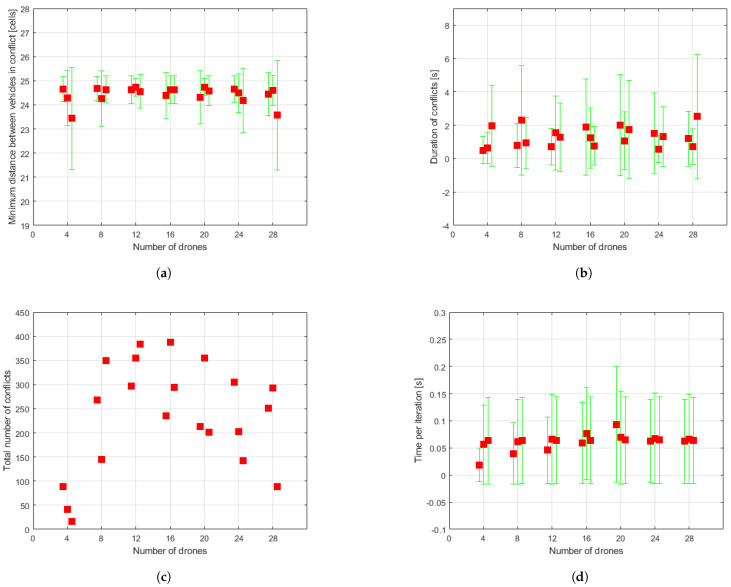

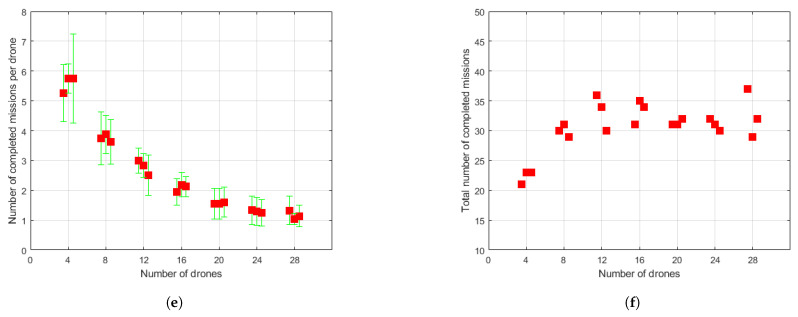
Statistical measures collected through simulations. Red squares represent mean values, while green ranges indicate mean absolute deviation. In the case of (**c**,**f**), red squares signify values directly collected from simulation. (**a**) Minimum distance found between vehicles in conflict. (**b**) Duration of conflicts. (**c**) Total number of conflicts. (**d**) Time per iteration. (**e**) Number of missions completed by each drone. (**f**) Total number of missions completed by the swarm.

The data resulting from the simulation runs are shown in Figure 8, Figure 9 and Figure 10. For each set of drones and for each run of the algorithm for the same set, some of the aforementioned measurements are presented directly through the value obtained in the simulation and others in the form of means either of the set of conflicts or of the set of drones, as appropriate. Besides, the dispersion of the obtained results is shown through mean absolute deviation measures. These values must be interpreted as they are: a measure of data dispersion. Negative values should not lead to misunderstandings. The data for each run carried out for each set are represented beside one another, around the vertical axis associated to the number of drones making up the swarm. Hereafter, some comments about the obtained data are expressed.

Measures related to the waiting time in depot required to start a new outbound mission (see Figure 8a) seem to increase proportionately with the number of drones that make up the swarm, as might be expected. However, the biggest swarm set of 28 drones result in a waiting time of about 2–3 min, not being a significantly long time. As for the waiting times to take off and begin the pertinent return mission (see Figure 8b), this measure does not seem affected by the number of drones of the set.

In relation to the real flight time/useful time ratios (Figure 9a,b) and the number of conflicts encountered in flight (Figure 9e,f), which are closely related, it seems that UAVs face more conflictive situations during their return missions to depot. Furthermore, the number of conflicts does not look to be directly linked to the number of vehicles in the swarm, except for the set of four UAVs which apparently results in fewer collision risk scenarios, as also shown in Figure 10c.

As for the distance traveled by the vehicles, results for both outbound (see Figure 9c) and return (see Figure 9d) missions and for every set of drones seem practically the same, since the goal position of the missions is chosen randomly regardless of any other factor.

Measures directly related to the resolution of conflicts yielded good results. The minimum distance found between vehicles in conflict (see Figure 10a) is kept propitiously at high values, around 24.5 cells. The duration of conflicts (see Figure 10b), although somewhat scattered, does not result in very long conflicts. Vehicles are stopped for an average of 2 s.

As shown in Figure 10d, the time required for each iteration of the control loop to run stays at about 0.06 on average, underlining the low computational load of the algorithm regardless of the number of drones making up the swarm.

Lastly, in relation to the missions completed by the drones, it can be appreciated in Figure 10e,f that the number of missions performed by each individual vehicle decreases along with the number of drones in the swarm. On the other hand, the number of missions carried out by the whole swarm is broadly the same for every set of drones, except for the smallest one of four drones which completes a few less.

## 4. Conclusions

The goal of this study was to design, implement and analyze a path planning and collision avoidance strategy for multi-UAV systems in 3D environments. The designed algorithm was implemented in MATLAB, conveniently and completely analyzing its performance through simulations.

This algorithm was divided into three stages. First, the 3D working environment simulating an urban environment was designed. In this environment, two UAV takeoff and landing zones, referred to as depots, were established. Subsequently, in this environment, the vehicles perform outbound and return missions to and from randomly chosen points of free space. The trajectories followed to perform these missions were calculated using the Fast Marching Square planning algorithm, which provides smooth, optimal paths in terms of both distance and time and, in addition, allows adding certain traffic constraints of interest with relative ease.

One of these restrictions consisted of establishing flight levels to guarantee a certain safety distance between UAVs and thus limit the number of potential conflicts, which are dealt with in the third stage of the algorithm: the collision avoidance strategy. Another conflict avoidance preventive measure considered was based on the special management of drone traffic in areas close to depots, the so-called exclusion zones, and those areas near the mission goal points. Conflicts that could not be avoided by these measures are dealt with through a simple resolution technique based on a distance check for conflict identification and a basic priority-based speed control to resolve them. The ability of the implemented solution to prevent, minimize, and resolve conflicts that could occur while the drones complete their missions was studied through several statistical measures.

From the obtained results, it is especially noteworthy that all the conflicts that occurred could be resolved, which demonstrates the great capacity of the algorithm to prevent deadlock scenarios. A considerable safety distance was guaranteed in every conflict. The standby times to which drones may be subjected, either due to conflict situations or for a safe takeoff, resulted in low waiting times. In addition, a good value of time per iteration was achieved, which opens up the possibility of applying the algorithm to a real system. In this sense and for future applications, the method should be suitably adjusted to the kinematic constraints of the vehicles involved and, as a consequence, the safety distances herein used could be tailored conveniently.

## Figures and Tables

**Table 1:** Method used to assign flight level according to flight direction.

Angle Ranges	$\left(-45^{\circ },45^{\circ }\right]$	$\left(45^{\circ },135^{\circ }\right]$	$\left(135^{\circ },225^{\circ }\right]$	$\left(225^{\circ },315^{\circ }\right]$
**Flight Level**	9	15	21	27
